# Effect of Fibronectin and Laminin on Compaction of Myoblast-Seeded Collagen Hydrogels

**DOI:** 10.3390/jfb17060299

**Published:** 2026-06-16

**Authors:** Sydnee T. Sicherer, Jasmine Guliani, Sandra A. Raju, Yash Parikh, Cassandra Martin, Jessi Pridmore, Katherine Coombs, Jonathan M. Grasman

**Affiliations:** Department of Biomedical Engineering, New Jersey Institute of Technology, Newark, NJ 07102, USA

**Keywords:** volumetric muscle loss, extracellular matrix, collagen hydrogels, tissue engineering, skeletal muscle

## Abstract

The extracellular matrix (ECM) regulates skeletal muscle development through biochemical signaling and mechanical interactions. While Matrigel supplementation is commonly used to enhance engineered muscle formation, the contribution of specific ECM proteins remain incompletely defined in 3D systems. Here, we evaluated the effects of laminin and fibronectin supplementation on myogenic differentiation in collagen type I hydrogels and assessed their influence on passive tissue compaction and alignment in 3D constructs. Two-dimensional collagen hydrogels supplemented with increasing concentrations (0–100 µg/mL) of laminin or fibronectin were screened to maximize the myoblast fusion index. These concentrations were incorporated into 3D myocyte-seeded hydrogels cultured between flexible posts to quantify passive compaction forces via cantilever mechanics. Fibronectin supplementation (10 µg/mL) resulted in significantly greater early post displacement and sustained passive compaction compared to laminin-supplemented and unsupplemented controls. Constructs cultured under tension between posts exhibited enhanced alignment, with fibronectin further increasing the proportion of fibers oriented within 0–20° of the tension axis. Together, these findings demonstrate that fibronectin enhances early passive compaction dynamics and tension-mediated alignment in collagen-based skeletal muscle constructs. These results provide insight into how specific ECM components influence 3D tissue organization and may inform the design of engineered muscle models for regenerative applications.

## 1. Introduction

Skeletal muscle injuries account for 77% of all healthcare visits in the United States and are a leading cause of disability and medical retirement amongst active soldiers [1]. Outside of combat, approximately 5.8 million surgeries are performed in the United States each year to address soft tissue injuries caused by tumor ablation, cosmetic procedures, and traumatic injury [2]. With many of these injuries occurring in the extremities, patients are often left with diminished mobility and a decreased quality of life [1]. While minor skeletal muscle injuries typically resolve over several weeks, muscle cannot repair itself if a large volume of tissue is removed [3]. Termed volumetric muscle loss (VML), this bulk loss of muscle tissue creates a wound that cannot regenerate and results in a permanent loss of function. Treatments such as physical therapy and autologous muscle transplantation aid in patient recovery but are limited by the size of the injury, tissue availability, and donor-site morbidity [3]. Engineered skeletal muscle tissues may provide alternative treatment options for VML that are not constrained by these limitations [4].

Crosstalk between myoblasts and their local extracellular matrix (ECM) plays a critical role in skeletal muscle growth and maturation. Changes in the concentration and composition of ECM proteins within skeletal muscle have been shown to regulate myoblast migration and differentiation and to drive specific myofiber subtypes [5,6,7]. “Bottom-up” tissue engineering approaches, in which myoblasts are embedded within hydrogels of defined chemical and fibrous composition, can support the formation of mature skeletal muscle prior to implantation. These strategies benefit from the incorporation of specific ECM components to direct targeted myoblast responses, particularly the formation of densely aligned, contractile myofibers, and have commonly been incorporated within biopolymers including fibrin or collagen hydrogels [8,9]. Notably, fabrication of skeletal muscle constructs typically requires the addition of ECM-rich supplements such as Matrigel to enhance myofiber maturation and contractile force production [10,11,12]. While its exact composition varies, Matrigel is composed of proteins and glycosaminoglycans found in the basement membrane—namely, laminin/enactin, fibronectin, type IV collagen, and heparan sulfate, among others [13,14]. Because of its batch-to-batch variability, it is unclear which specific proteins may aid in tissue maturation and development, and because of its oncogenic origins, it is unlikely to be FDA-approved for usage in humans. Exploring the effect of individual ECM constituents on skeletal muscle development can bypass the reliance on Matrigel in fabricating biomimetic muscle constructs. While it is common to add specific basement-membrane proteins to hydrogels, systematic evaluation of individual ECM protein contributions to tissue organization and alignment in collagen-based 3D muscle constructs remains limited [10,11,12]. Since many of these molecules play key roles in mediating myofiber behavior and maturation and are expressed at different points in muscle development, we developed a non-invasive, purpose-built device that is highly sensitive to tissue forces to allow us to explore the effects that these proteins have on tissue morphology and contractility [5,6,10,15,16,17,18,19,20].

In this study, we hypothesized that the addition of proteins present in native muscle ECM would result in biomimetic tissues with enhanced myofiber alignment and organization. To achieve this goal, we first utilized two-dimensional collagen gels as a screening platform to identify ECM protein concentrations associated with enhanced myogenic differentiation. These concentrations were subsequently incorporated into three-dimensional collagen constructs to evaluate whole-tissue behaviors, including passive compaction and alignment under tension in our contractile force indicator (CFI) device. Type I collagen was selected as the bulk material because of its abundance in native skeletal muscle ECM. Fibronectin was selected due to its prevalence in early skeletal muscle development, and laminin was selected due to its prevalence in mature skeletal muscle tissue [5,21]. A 3D-printed CFI device consisting of a mobile polydimethylsiloxane (PDMS) post and an immobile biocompatible resin post was developed to noninvasively determine compaction force within tissues throughout the culture period. Skeletal muscle constructs were cultured around the two posts of the device, compaction force was calculated by measuring displacement of the mobile post over time, and after culture, the overall tissue structure and myofiber alignment were determined to elucidate the effect that these proteins have on skeletal muscle mimetic organization and development. These results describe the relative effect of several ECM proteins on skeletal-muscle-construct passive force production and organization and will aid in the creation of skeletal muscle constructs to study muscle biology and develop alternative treatments for VML injuries.

## 2. Materials and Methods

### 2.1. Cell Culture

Immortalized C2C12 myoblasts (CRL-1772, ATCC, Manassas, VA, USA) were cultured in growth medium (GM; DMEM/F12 (Gibco, Waltham, MA, USA) supplemented with 10% fetal bovine serum (FBS, Gibco) and 1% antibiotic–antimycotic solution (Gibco)) in T75 tissue culture flasks at 37 °C under 5% CO_2_ and passaged at 60–70% confluence. Routine cell passaging was conducted using 0.05% trypsin (Gibco) and manual agitation. To amplify cell numbers for the described studies, myoblasts were expanded to 95% confluence prior to trypsinization and immediate use in experiments.

### 2.2. Determination of ECM Concentrations to Enhance Myoblast Differentiation Within 2D Hydrogels

To determine the optimized concentration of ECM constituents to enhance myoblast differentiation, 2D collagen gels were supplemented with varying concentrations of ECM proteins and seeded with C2C12s. Collagen type I (TeloCol-10, Advanced Biomatrix, San Diego, CA, USA) was combined with 10X phosphate-buffered saline (PBS) and 1 N sodium hydroxide (NaOH) per the manufacturer’s instructions to produce hydrogels with a final collagen concentration of 3 mg/mL. C2C12s were combined with GM (final concentration: 100,000 cell/mL) and mixed with fibronectin (Corning, Bedford, MA, USA) or laminin (Corning) in order to create tissues with final concentrations of 0, 1, 10, or 100 µg/mL of either protein. Collagen I and cell/protein solutions were mixed together, seeded in 48-well plates, and incubated at 37 °C for one hour to polymerize. After polymerization, differentiation medium (DM; DMEM/F12 supplemented with 2% heat-inactivated horse serum (Gibco), 1% insulin–transferrin–selenium (Gibco), and 1% antibiotic–antimycotic solution) was added to each well, and the medium was changed daily. After seven days of culture, samples were fixed in 4% paraformaldehyde for one hour and prepared for immunostaining.

### 2.3. Creation of Contractile Force Indicator (CFI) Device

**Figure 1:**
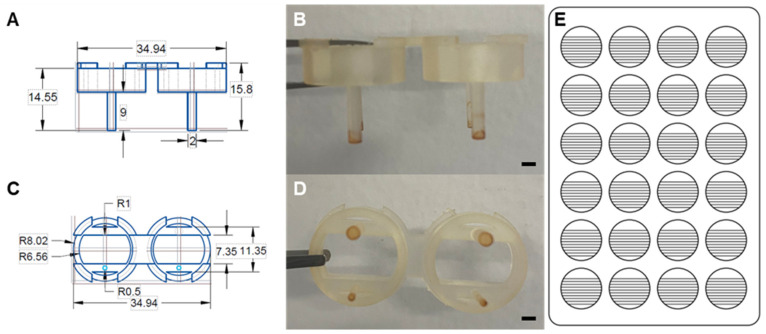
Overview of the contractile force indicator (CFI) device. (**A**,**C**) Technical drawings and (**B**,**D**) macroscopic images of the CFI device (Scale = 2 mm). (**E**) Schematic of the layout of the laser-cut ruler, which was positioned beneath the well plate during culture and imaging.

**Figure 2:**
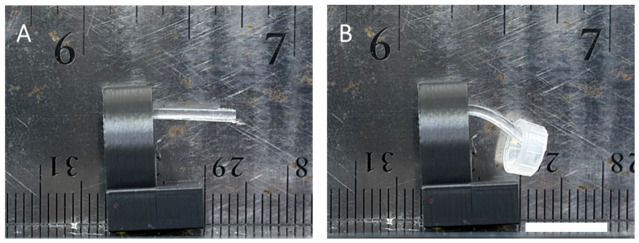
Physical validation of PDMS post stiffness by measuring cantilever deflection. Representative side-view images of a PDMS post (**A**) before and (**B**) after application of a 45 mg calibration mass to the free tip (background is a ruler to visualize scale/displacement). Angular deflection (Δθ) was measured from calibrated images and used to derive tip displacement. Young’s modulus was calculated using the Euler–Bernoulli cantilever beam equation. Scale bar = 1 mm.

The CFI device utilized in this study was created in CAD and 3D-printed using Formlabs biomed amber resin (Formlabs, Boston, MA, USA) (Figure 1). Devices were rinsed in isopropyl alcohol and cured as per the manufacturer’s instructions. The ruler was designed in CAD based on measurements of the bottom of a 24-well plate and was laser-engraved onto clear 1.5 mm acrylic using a speedy Flexx 400 (Trotec, Marchtrenk, Austria) (Figure 1E). The mobile PDMS post was created from a 15:1 ratio of elastomer base to curing agent (Sylgard, St. Louis, MO, USA), which, after mixing, was incubated under vacuum for one hour to remove bubbles. The de-gassed PDMS was poured over an aluminum mold with 0.5 mm radius holes for the posts and, again, incubated for 1 h under vacuum to remove air bubbles and to draw the PDMS completely into the mold. The PDMS-loaded aluminum mold was cured for one hour at 70 °C. After curing, posts were removed from the mold, fastened to the CFI device using Dot Loctite Ultra Gel Control Super Glue (Henkel Corporation, Düsseldorf, Germany), and autoclaved prior to usage. A subset of PDMS was cast into strips for uniaxial tensile testing to determine the Young’s modulus by stretching samples at a strain rate of 10 mm/min until fracture. Strain was normalized to the gauge length, and stress was calculated by dividing the force by the initial cross-sectional area of the PDMS strip. To validate the empirically derived stiffness values used in force calculations, a subset of posts from two independent fabrication batches (n = 4 per batch) were evaluated across a total of eight devices. Posts were suspended in a custom setup, cantilever deflection was determined under a known load (45 mg) (Figure 2), and Young’s modulus was calculated using the Euler–Bernoulli cantilever beam equation:

$$E=\frac{\left(F\times L^{3}\right)}{\left(3\times I\times \delta \right)}$$

where deflection (δ) is derived from the measured angular deflection (Δθ) of each post before and after loading (δ = L·sin(Δθ)), F is the applied load (4.41 × 10^−4^ N), L is the length of the post, I is the area moment of inertia (πd^4^/64), and d is the diameter of the post. The deflection-derived modulus (1.025 ± 0.147 MPa, N = 8 independent devices) agreed with the tensile-test value to within 2.4%, confirming the accuracy of the material property used in force calculations. Inter-device variability was 14.3% across the two batches (Table 1). Devices were inverted within a P100 dish, and hanging drops of Sulfo-SANPAH (MilliporeSigma, Burlington, MA, USA) were positioned on the lid to immerse the tips of both posts. The P100 dish was placed in a UV oven (VWR Avantor, Radnor, PA, USA) and cured for five minutes at 320–350 nm to deliver 120,000 µJ. This process was repeated once with fresh Sulfo-SANPAH hanging drops, and any uncured Sulfo-SANPAH was washed away with sterile DI water. Devices were transferred into a 24-well plate for immediate use.

### 2.4. Fabrication of 3D Muscle Constructs with Optimized ECM Protein Concentrations

**Figure 3:**
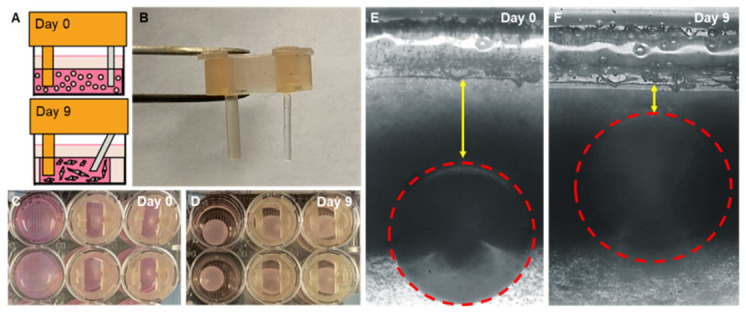
Overview of experimental method to determine passive contraction force of myoblast-seeded hydrogels. (**A**) Cartoon depicting how the mobile post is displaced as the gel contracts. (**B**) Representative image of a fully assembled device. Representative images of gels with and without the contraction device (**C**) at the start of culture and (**D**) after 9 days of culture. (**E**) Representative overlaid images of the post and ruler at the start of culture and (**F**) after 9 days of culture. The deflecting post is indicated with the dashed red circle, and the distance between the post and the ruler is indicated by the yellow line.

Collagen I hydrogels (final concentration: 3 mg/mL) were fabricated as described above and supplemented with optimized concentrations of ECM proteins from the 2D experiments (100 µg/mL laminin or 10 µg/mL fibronectin) and a final C2C12 concentration of 2 million cells/mL. Collagen-cell solutions were vigorously mixed to ensure that all constituents were combined evenly and subsequently pipetted into each well, surrounding the two posts of the CFI device (Figure 3A). Cell-laden hydrogels mixed with 20% (*v*/*v*) Matrigel and hydrogels without any ECM proteins served as positive and negative controls, respectively. To account for the effect that the device, itself, may have had on tissue alignment and fiber formation, we cultured hydrogels in well plates without any devices (Figure 3C,D). After incubation at 37 °C to ensure polymerization, samples were carefully detached from the walls of the wells and cultured in GM for three days, followed by seven days in DM, for a total of ten days in culture prior to fixation with 4% paraformaldehyde PFA for 1.5 h. After fixation, samples were rinsed with 1X PBS and incubated in 30% (*w*/*v*) sucrose (Fisher Scientific, Waltham, MA, USA) in PBS overnight, transferred into OCT, flash-frozen in liquid nitrogen, and cryosectioned at a thickness of 10 µm. Samples were embedded in cross-section such that both posts could be visualized in each section.

### 2.5. Quantifying the Effect of ECM Protein Supplementation on 3D Tissue Compaction

Tissue compaction was quantified by measuring the displacement of the mobile post daily. Displacement was defined as the location of the post with respect to a reference ruler that was attached to the bottom of the 24-well plate prior to initiation of the culturing period (Figure 3E,F). Posts and the reference ruler were imaged with a Keyence BZ-X810 fluorescent microscope (Keyence Corporation of America, Itasca, IL, USA), and the position of the post with respect to the reference ruler was measured using ImageJ (Version 1.54t, NIH, Bethesda, MD, USA). Displacement was calculated as the change in distance at Day X from the initial distance recorded immediately after collagen polymerization on Day 0 (Day 0–Day X), (Figure 3E,F). Compaction of constructs cultured without the CFI device were calculated using diameter measurements from macro-images of the constructs using the same formula as above: (Day 0–Day X). Tissue compaction force was quantified using cantilever mechanics and was calculated with the following formula:

$$Force\left(mN\right)=\frac{\left(3\times deflection\times E\times \pi r^{4}\times 1000\right)}{\left(4\times l^{3}\times 1000^{2}\right)}$$

where E is the Young’s modulus of PDMS, which was empirically determined to be 1.05 MPa; r is the radius of the post (0.50 mm); l is the length of the post (10.00 mm); and deflection is the measured post displacement.

### 2.6. Immunostaining

After fixation, 2D samples were stained for myosin heavy chain (MyHC) and counterstained with 4′,6-diamidino-2-phenylindole (DAPI) to calculate the fusion index. Fixed samples were permeabilized with 0.1% (*w*/*v*) Triton-X100 (Fisher Scientific) in PBS for twenty minutes, rinsed twice in 0.05% (*w*/*v*) Tween-20 in PBS (PBST, Fisher Scientific), and blocked with 5% (*w*/*v*) bovine serum albumin (BSA, Fisher Scientific) in PBST for 30 min. Immediately after blocking, the solution was replaced with a primary antibody targeting MyHC (MF-20, 1:20, Hybridoma Bank, Iowa City, IA, USA), mixed in 1% (*w*/*v*) BSA/PBST, and incubated for two hours at room temperature. After incubation, samples were washed twice with 0.05% PBST, incubated with species-matched AlexaFluor 488 (1:500, Fisher Scientific) and DAPI (1:1000, Invitrogen, Rockford, IL, USA), mixed in 1% (*w*/*v*) BSA/PBST, and incubated for 45 min at room temperature. Samples were subsequently washed thrice with 1X PBS and visualized with a Keyence BZ-X810 microscope using a 10X objective. The extent of the ECM protein concentration on tissue maturation was quantified by calculating the fusion index in ImageJ. The fusion index was defined as the percentage of nuclei within MyHC-positive fibers normalized to the total number of nuclei per image. Protein concentrations that resulted in the highest fusion index were utilized in the creation of 3D tissues.

To determine myofiber arrangement within 3D samples, tissues were cryosectioned, and sections were immunostained for myosin heavy chain (MyHC). Sections were permeabilized with 0.1% Triton-X100/PBS for twenty minutes, rinsed twice in 0.05% PBST, and blocked with BSA for 30 min; then, solutions containing primary antibody (MF-20) were incubated on samples for two hours. Samples were subsequently washed twice in Coplin jars containing 0.05% PBST. After washing, samples were incubated with species-matched Alexa-Fluor 594 secondaries (1:500, Invitrogen) and Alexa-Fluor 488 phalloidin (1:500, Invitrogen) for 45 min. Samples were washed thrice in 1X PBS in clean Coplin jars, and DAPI-containing mounting medium (Abcam, Waltham, MA, USA) was added dropwise to each sample and sealed in place with a coverslip. Stained sections were visualized with a Keyence BZ-X810 microscope with appropriate filter cubes.

### 2.7. Characterization of Myofiber Alignment in 3D Tissues

**Figure 4:**
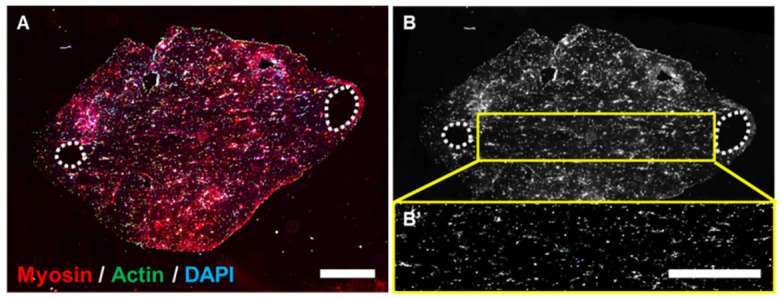
Methodological workflow to measure myoblast alignment. (**A**) Representative image of a cryosectioned hydrogel. White dashed circles represent the positions of the posts. Scale = 2000 µm. (**B**) Images were converted to 8-bit and rotated in ImageJ so that the angle between the two posts was zero. The yellow box indicates the selected region that was analyzed for alignment (highlighted in (**B’**), scale = 2000 µm).

To determine the alignment of myofibers within 3D hydrogel constructs, stained sections were analyzed in Fiji (ImageJ plugin). Construct sections were fully imaged using a 10X objective and merged into one composite image (Figure 4A) that was rotated in Fiji to ensure that the two posts of the device were level with the *x*-axis of the image. After rotation, images were converted to an 8-bit format (Figure 4B). The region of alignment between the posts was selected with the rectangle tool in Fiji, and fiber alignment was quantified using the “directionality” tool (Figure 4B’). Within this tool, the selected method was “Fourier components”, and the number of N-bins was set to 181 to produce data organized from −90° to 90°. Measurements were produced by selecting the “display table” output option. As there is symmetry in fiber alignment across 0°, data were aggregated to 0–90°, binned in 10° increments, and plotted in a histogram presenting the average frequency of each bin size [22].

### 2.8. Statistics

Data are presented as mean ± standard error unless otherwise stated. Sample sizes are indicated as the total number of analyzed hydrogel constructs, followed by the number of independent experiments conducted. Statistical analyses were performed using a one-way ANOVA, with *p* < 0.05 indicating significant differences between groups according to SPSS software (Version 27, IBM^®^, Armonk, NY, USA). For post hoc analysis, Tukey’s post hoc test was performed to determine significant differences between experimental groups using an overall significance level of *p* < 0.05. For time-course data (post displacement, hydrogel diameter, and calculated compaction force), independent statistical comparisons were performed between experimental groups at each time point using one-way ANOVA with Tukey post hoc analysis.

## 3. Results

### 3.1. ECM Concentrations Associated with Increased Fusion Index in 2D Collagen Gels

**Figure 5:**
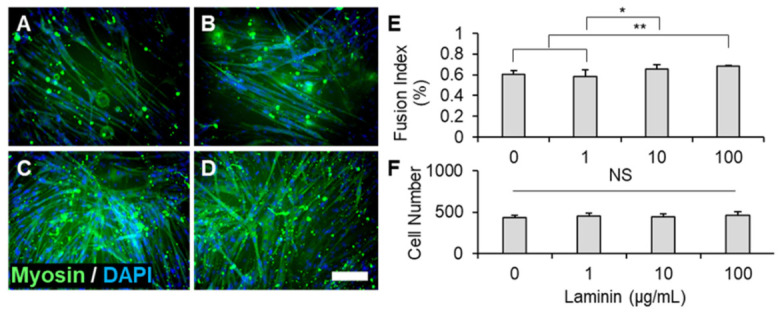
Representative images of collagen hydrogels supplemented with (**A**) 0, (**B**) 1, (**C**) 10, or (**D**) 100 µg/mL of laminin. Scale = 500 µm. Quantification of (**E**) the fusion index and (**F**) cell number per region of interest. Brackets indicate statistical significance between corresponding groups as determined by one-way ANOVA with Tukey post hoc analysis (* *p* < 0.05, ** *p* < 0.01; N ≥ 9 hydrogels across 3 independent experiments). NS: no significance.

To determine the concentration of each ECM protein that resulted in the highest fusion index after 7 days of differentiation, 3 mg/mL collagen gels were supplemented with 0, 1, 10, or 100 µg/mL of laminin or fibronectin and seeded with C2C12 myoblasts. After 7 days in culture, the fusion index of each treatment group was measured using Fiji. Representative images of laminin-supplemented hydrogels revealed that while all treatment groups supported myofiber formation (Figure 5A–D), there were qualitatively more myofibers present with 10 µg/mL (Figure 5C) and 100 µg/mL (Figure 5D) of laminin. No consistent differences in fiber orientation were observed across laminin concentrations in this static 2D system. Quantitatively, the fusion index was significantly higher with 100 µg/mL of laminin than 0 or 1 µg/mL laminin, and the fusion index with 10 µg/mL laminin was significantly higher than that with 1 µg/mL of laminin (Figure 5E). Importantly, the cell number within each treatment group did not change (Figure 5F), suggesting that cell number and proliferation were not impacted by laminin concentration and that that 100 µg/mL laminin was associated with a significantly higher fusion index relative to the 0 and 1 µg/mL conditions.

**Figure 6:**
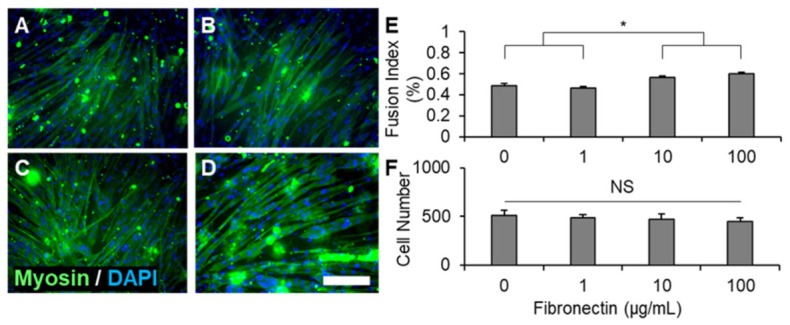
Representative images of collagen hydrogels supplemented with (**A**) 0, (**B**) 1, (**C**) 10, or (**D**) 100 µg/mL of fibronectin. Scale = 500 µm. Quantification of (**E**) the fusion index and (**F**) cell number per imaged region. Brackets indicate statistical significance between corresponding groups as determined by one-way ANOVA with Tukey post hoc analysis (* *p* < 0.05; N ≥ 9 hydrogels across 3 independent experiments). NS: no significance.

Representative images of fibronectin-supplemented hydrogels revealed that increasing concentrations of fibronectin qualitatively enhanced myofiber formation, particularly in comparison with collagen-only hydrogels (Figure 6A–D). Despite increases in the number of myofibers, no myofiber alignment was observed. Quantitatively, the fusion index in collagen hydrogels was significantly higher with concentrations of 10 µg/mL and 100 µg/mL fibronectin relative to 0 µg/mL and 1 µg/mL of fibronectin (Figure 6E). Similar to results with laminin-supplemented hydrogels, there were no differences in the cell number between fibronectin concentrations, implying that the cell concentration was consistent across all fibronectin concentrations and that any differences in the fusion index did not result from changes in proliferation.

From these data, a final concentration of 100 µg/mL of laminin was selected for 3D studies, as this concentration resulted in a significantly higher fusion index when compared to unsupplemented gels and gels supplemented with 1 µg/mL of laminin (Figure 5). While 10 µg/mL laminin also significantly elevated the fusion index relative to 1 µg/mL, it was not significantly greater than the unsupplemented control, whereas a significant difference was observed with 100 µg/mL. In the absence of a statistically significant difference between 10 and 100 µg/mL, we selected the higher concentration (100 µg/mL) to maximize the potential for ECM-mediated enhancement in the transition to 3D culture. A final concentration of 10 µg/mL was selected for fibronectin for 3D studies, as this concentration resulted in a significantly greater fusion index than unsupplemented gels and gels supplemented with 1 µg/mL of fibronectin (Figure 6). Furthermore, there were no differences between the 10 µg/mL and 100 µg/mL concentrations for fibronectin, suggesting a plateau in the dose-dependent effect of fibronectin on the fusion index.

### 3.2. Fibronectin Improves Compaction Force in Our 3D Model

To determine the effect of an optimized ECM protein concentration on 3D biomimetic tissue compaction and myofiber organization, ECM myoblast-supplemented collagen hydrogels were seeded within CFI devices such that the hydrogel was in contact with both the mobile and immobile posts (Figure 3A,B). Hydrogels supplemented with 20% Matrigel or no exogenous ECM proteins served as positive and negative controls, respectively. Displacement of the mobile post and the tissue diameter of constructs cultured without devices were measured daily (Figure 3C–F), and compaction force was calculated from displacement values using cantilever mechanics.

**Figure 7:**
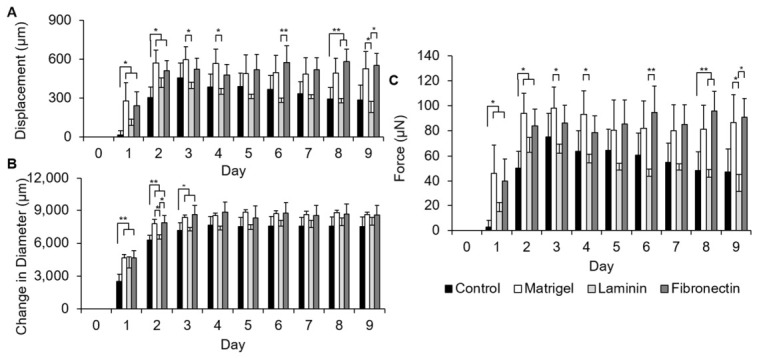
(**A**) Quantification of the average displacement of the mobile post over nine days of culture. (**B**) Quantification of the change in hydrogel diameter when cultured without the CFI device over nine days of culture. (**C**) Quantification of compaction force within hydrogels as measured by displacement values in (**A**) using cantilever mechanics. Brackets indicate statistically significant differences between corresponding conditions as determined by one-way ANOVA with Tukey post hoc analysis (* *p* < 0.05, ** *p* < 0.01; N ≥ 13 hydrogels across 7 independent experiments).

Hydrogels supplemented with Matrigel and fibronectin supported significantly greater post displacement than unsupplemented controls during the first 48–72 h of culture (Figure 7A). By day three, all samples supported similar amounts of post displacement, except for laminin-supplemented hydrogels. Fibronectin-supplemented hydrogels supported continued increases in displacement throughout the culture period, although they qualitatively reached a plateau around day 3 (524 ± 84 µm) and supported maximum post displacement on day 8 (583 ± 93 µm). No hydrogel relaxation, which was defined as a decrease in post displacement between consecutive time points, was observed in fibronectin-supplemented hydrogels. Matrigel-supplemented hydrogels also reached peak displacement on day 3 (596 ± 102 µm) and exhibited qualitative hydrogel relaxation after day 3. Laminin hydrogels supported significantly less post displacement throughout the study, reaching maximum displacement on day 2 (380 ± 73 µm) and subsequently exhibited hydrogel relaxation for the remaining seven days in culture, particularly on days 3, 4, 6, and 9. Control hydrogels supported maximum displacement on day 3 (457 ± 114 µm) and also exhibited qualitative relaxation, particularly at day 8.

To separate the contributions of the CFI device on tissue compaction from ECM composition, muscle mimetics were similarly cultured without a device for 9 days, and the change in diameter was measured daily (Figure 7B). Similar to results obtained with the CFI device, Matrigel- and fibronectin-supplemented hydrogels contracted rapidly between days 0 through 4 as compared to control and laminin-supplemented hydrogels, which exhibited slower and significantly less compaction. Compaction of Matrigel- and fibronectin-supplemented hydrogels was significantly greater than that supported by control hydrogels for the first 3 days of culture and qualitatively supported the highest amounts of compaction for the remainder of the experiment.

Tissue compaction forces reflect observations of mobile post displacement recorded with the CFI device (Figure 7C). Fibronectin- and Matrigel-supplemented hydrogels generated significantly larger forces than control hydrogels on days 1, 2, and 8 of the 9-day study. On day 1, both fibronectin- and Matrigel-supplemented constructs produced approximately thirteenfold higher forces compared to control hydrogels, while by day 2, compaction force production remained elevated by roughly twofold relative to control hydrogels. By day 3, all samples, aside from laminin, exhibited similar amounts of compaction force production, and it was noted that Matrigel produced significantly more forces than laminin on days 4 and 9. Laminin supported less compaction force production than all other tested ECM constituents. Pairwise comparisons revealed no significant difference in tissue compaction between fibronectin- and Matrigel-supplemented constructs at any time point throughout the culture period, suggesting that fibronectin alone is sufficient to recapitulate the compaction force dynamics supported by Matrigel in this system. During the first two days of culture, both fibronectin- and Matrigel-supplemented constructs produced significantly greater post displacement than unsupplemented constructs. Fibronectin-supplemented constructs did not result in any of the tissue relaxation that was observed with control and laminin constructs, resulting in significantly greater compaction forces at later time points that also reflected the sustained remodeling capacity of fibronectin relative to laminin over the full culture period.

### 3.3. Fibronectin Supplementation Increases Myofiber Alignment in 3D Tissues

**Figure 8:**
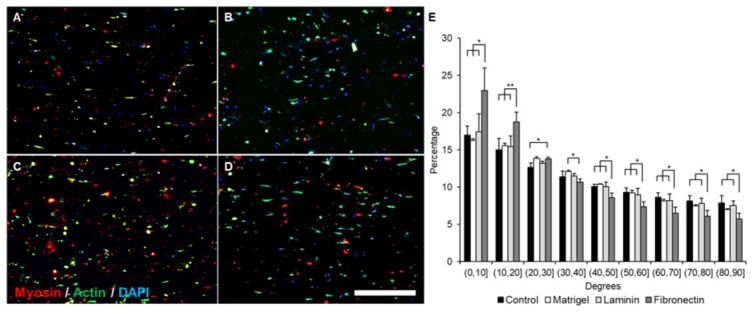
Myofiber alignment within hydrogels cultured in our CFI device. Representative images of (**A**) the control and (**B**) Matrigel-, (**C**) laminin-, and (**D**) fibronectin-supplemented hydrogels. Scale = 600 µm. (**E**) Histogram of the percentage of myofibers oriented within the indicated degree range of the center line between the two posts. The more aligned a tissue, the higher the percentage of fibers that fall within 0–20°. Brackets indicate statistically significant differences between corresponding conditions as determined by one-way ANOVA with Tukey post hoc analysis (* *p* < 0.05, ** *p* < 0.01; N ≥ 3 hydrogels across 7 independent experiments).

To determine the contribution of ECM proteins to myofiber alignment, ECM-supplemented hydrogels were cultured in the CFI device for 9 days, fixed, cryosectioned, and immunostained against MyHC, actin, and nuclei. All samples cultured with the CFI device displayed some level of cell alignment and elongation parallel to the axis of tension between the posts. There were similar cell concentrations and cell–cell spacing across all treatment groups, suggesting that proliferation and migratory behaviors were not altered in these culture environments (Figure 8A–D). Qualitative observations demonstrate that fibronectin supplementation supported more elongated cell morphologies than other cell-laden hydrogels. Amongst tissues cultured with the CFI device, alignment was significantly enhanced in fibronectin-supplemented hydrogels (Figure 8E). Fibronectin supplementation supported greater percentages of myofibers angled between 0 and 20° with respect to the centerline between the two posts than all other hydrogel conditions. Samples supplemented with fibronectin exhibited approximately 28% more fibers within 0–10° of the centerline than other tested variables, and approximately 27% more fibers within 10–20° of the centerline. The percentage of myofibers that showed minimal preferential alignment to the centerline (30–90° cell angles) was also significantly lower for fibronectin-supplemented hydrogels compared to all other treatment groups, indicating less fiber deviation from the centerline.

**Figure 9:**
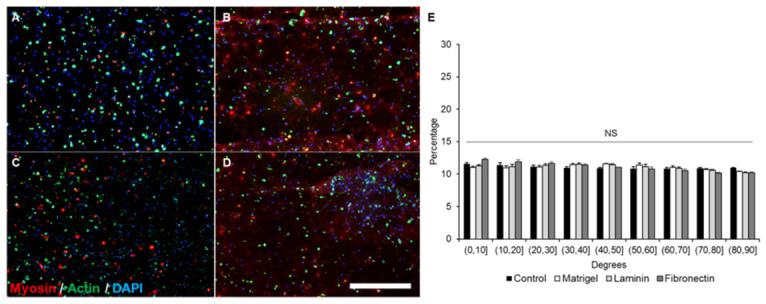
Myofiber alignment within hydrogels cultured in well plates without the CFI device. Representative images of (**A**) the control, (**B**) Matrigel, (**C**) laminin, and (**D**) fibronectin. Scale = 600 µm. (**E**) Histogram of the percentage of myofibers oriented within the indicated degree range of the center line. The more aligned a tissue, the higher the percentage of fibers that fall within 0–20° of the centerline. No significance (NS) was detected between any conditions as determined by one-way ANOVA with Tukey post hoc analysis (N ≥ 3 hydrogels across 7 independent experiments).

Cell-laden hydrogels cultured without CFI devices exhibited similar morphology across all experimental conditions (Figure 9A–D). There were no differences in cell concentration or cell–cell spacing when comparing cultures with and without the CFI device. In general, cells cultured without the CFI device appeared to be spherical and did not show any preferential elongation or alignment. Cell alignment was evenly dispersed throughout the entire construct (0–90° of the centerline, Figure 9E). No significant or qualitative differences in alignment were observed between experimental groups, implying the addition of ECM proteins did not impact alignment or the arrangement of cells without the CFI device. This distribution is significantly different than the distribution of hydrogels cultured with the CFI device, demonstrating that the two-post system and fibronectin supplementation are responsible for the significant changes in alignment observed in these studies.

## 4. Discussion

The goal of this study was to identify which constituent proteins found in the skeletal muscle ECM are responsible for driving myofiber alignment and tissue organization by (1) optimizing the concentrations of ECM proteins in collagen hydrogels to maximize myofiber formation, (2) determining the impact of these proteins on muscle mimetic organization, and (3) measuring the tissue compaction forces of these mimetics utilizing our CFI device. To achieve these goals, we explored the effects of increasing concentrations of laminin and fibronectin on the fusion index in 2D and applied the results from these optimization studies to our 3D CFI device experiment to determine how each ECM protein affected biomimetic tissue compaction and cell alignment. Our findings support a significant relationship between ECM composition and biomimetic tissue development. In the first part of this study, we determined that concentrations of 10 µg/mL fibronectin and 100 µg/mL laminin resulted in the highest degree of myofiber maturation in 2D. These results reflect existing literature; fibronectin and laminin are present in the basal and reticular lamina of skeletal muscle and are associated with myoblast maturation, proliferation, and differentiation. Differences in the fusion index as a result of protein concentration have also been observed in aged skeletal muscle and may reflect changes in local ECM composition. For example, the overexpression of laminin and underexpression of fibronectin have been associated with reduced regenerative capabilities in aged skeletal muscle [23]. Independent work supplementing laminin into 3D fibrin scaffolds reported that higher concentrations of laminin resulted in decreased myoblast maturation and increased cell proliferation [10,11]. In contrast, we observed a positive relationship between laminin concentration and myofiber maturation, with no changes in cell proliferation. Separately, we observed a plateau in the fusion index for hydrogels supplemented with 10 µg/mL of fibronectin. The plateau in the fusion index observed at higher fibronectin concentrations may reflect the saturation of fibronectin-binding integrins, particularly α5β1, which mediates fibronectin-mediated adhesion and differentiation signaling in myoblasts. When the ligand density exceeds the available integrin-binding capacity, additional ECM molecules may not further enhance downstream signaling. Similar integrin-dependent thresholds have been reported in studies examining fibronectin-mediated myogenesis and mechanotransduction [24,25,26]. It should be noted that 2D and 3D culture environments differ substantially in ECM protein presentation, diffusion kinetics, and cell-matrix interaction geometry, and the concentrations identified through 2D screening should therefore be regarded as informed starting points rather than definitive optima for the 3D system; systematic concentration optimization within the 3D collagen hydrogel environment itself represents an important direction for future work. In summary, by independently incorporating each ECM protein into skeletal muscle constructs, we were able to associate whole-tissue behaviors with distinct components of the native ECM. Our data indicate that fibronectin enhances tissue compaction, compaction force production, and myoblast alignment in hydrogels held in tension between two posts over control and laminin-supplemented hydrogels. Interestingly, while compaction force production was similar between fibronectin- and Matrigel-supplemented hydrogels, only fibronectin-supplemented hydrogels enhanced alignment.

It was interesting to note that fibronectin supplementation supported compaction similarly to Matrigel-supplemented constructs. Our data demonstrate that fibronectin supports earlier and more sustained compaction force production than laminin supplementation and our negative control in the first four days of culture. These values are similar to those from constructs supplemented with Matrigel, which is the gold standard for the fabrication and culture of muscle mimetics [12]. Although Matrigel contains large amounts of laminin, it also includes collagen IV, fibronectin, entactin, and growth factors that may contribute synergistically to compaction behavior. The similarity between fibronectin- and Matrigel-supplemented constructs suggests that fibronectin may recapitulate specific aspects of Matrigel-mediated compaction within collagen-based systems. Samples supplemented with Matrigel or fibronectin and cultured without the CFI device exhibited earlier (0–4 days) and significantly more compaction than control tissues, suggesting that this observed change in the rate and extent of compaction is not entirely dependent on tension. Previous studies using fibrin and collagen hydrogels supplemented with varying concentrations of Matrigel demonstrated that construct diameter was influenced by both the base material and the proportion of Matrigel added. Notably, 10% Matrigel supplementation in collagen hydrogels supported greater compaction and smaller diameters than 10–40% Matrigel supplementation in fibrin hydrogels [12]. In conjunction with our findings, these results suggest that tissue compaction and force production are governed by the interplay between the bulk material and the specific exogenous proteins incorporated. Among the proteins we evaluated, fibronectin emerged as a key contributor to this relationship. It should be noted that mechanical characterization of the ECM-supplemented hydrogels was not performed in this study. While stiffness differences between the fibronectin- and laminin-supplemented groups are anticipated to be modest, given the consistent collagen backbone and low supplementation concentrations, the incorporation of 20% *v*/*v* Matrigel in our positive control represents a more substantial addition to the hydrogel and may introduce meaningful differences in bulk material properties [27]. We therefore cannot exclude the possibility that stiffness differences may have contributed to the compaction behavior observed in the Matrigel group, and mechanical characterization of all hydrogel formulations is an important direction for future work.

Changes in tissue compaction and compaction force production may arise as a consequence of intracellular signaling induced by integrin binding. Laminin and fibronectin interact with different integrins: laminin binds with α6β1 and α7β1 integrins, while fibronectin binds to α4β1 and α5β1 integrins [26,28]. The α7β1 integrin serves as a critical linkage point between laminin in the ECM and the actin cytoskeleton of skeletal muscle fibers, with mutations in this integrin underlying the majority of myopathies, highlighting the essential role of laminin-α7β1 binding in myofiber adhesion and cytoskeletal integrity. Furthermore, α7β1 has been implicated in protein upregulation and the maintenance of myoblast homeostasis in response to mechanical loads [26]. In the present study, fibronectin-treated constructs supported measurable tissue compaction that was absent in laminin-treated constructs. This differential response may not be attributed to changes in internal integrin-cell signaling but, rather, to the binding of α4β1 and α5β1 integrins to fibronectin, which is proposed to enhance mechanical anchorage and promote enmeshing between cells and the surrounding collagen matrix [29,30].

The differences in compaction may also reflect different patterns in developing skeletal muscle. Integrins associated with fibronectin, as well as the presence of fibronectin itself, are observed earlier in embryonic muscle development than laminin and are associated with cell adhesion and migration. Laminin, which is deposited later in development, is associated with further structural stabilization and maturation of the tissue [19]. The consistently low amount of compaction in laminin-supplemented hydrogels may have been a consequence of seeding immature cells into an environment that was selective for maturation and not proliferation. It should be noted that the forces measured in this study represent passive compaction forces arising from cell-mediated hydrogel remodeling throughout culture and are distinct from active contractile forces, which would require electrical or mechanical stimulation of mature myotubes to assess.

While the ultimate rate of compaction is similar between tissues cultured with and without the CFI device, myofiber alignment is only observed in tissues cultured under tension. Myofiber alignment under tension has been explored in depth and is a common method of inducing alignment in 3D skeletal muscle tissue models [31]. These results imply a potentially synergistic effect on tissue organization between ECM protein composition and mechanical force. Studies exploring the effect of strain on skeletal muscle myoblasts have revealed further relationships between integrin expression, the ECM proteins that these integrins specifically interact with, and their combined response to mechanical stimuli. The binding of integrins to specific ECM proteins acts as a mechanism for both sensing the presence of these proteins and facilitating cell adherence to the ECM. The application of external mechanical forces to integrins is associated with improved binding affinity, integrin clustering, and changes in stress fiber assembly within the cell itself [5,17,26,28]. We observed less tissue relaxation in constructs cultured with the CFI device and supplemented with Matrigel or fibronectin. While pure collagen hydrogels and laminin-supplemented hydrogels exhibited relaxation beginning around day five in culture, this behavior was not observed with Matrigel or fibronectin supplementation. Studies exploring the effect of fibronectin on other cell types have observed similar phenomena; tissues cultured with fibronectin exhibit compaction at earlier time points and maintain this level of compaction as compared to non-treated controls [32,33]. Ultimately, the inclusion of both fibronectin and tension appears to be beneficial in creating compact, aligned tissues.

## 5. Conclusions

In summary, this study sought to elucidate the role of specific ECM proteins in controlling skeletal muscle tissue compaction and organization. Our data suggest that fibronectin supplementation may be sufficient to produce highly compact skeletal muscle tissues. The addition of fibronectin supported sustained hydrogel contraction that did not exhibit relaxation throughout culture, replicating the behavior of Matrigel-supplemented hydrogels. The CFI device utilized in this study provided further evidence that tension directs myofiber alignment. Specifically, myofibers were randomly organized when cultured without the CFI device, even in the presence of exogenous ECM proteins. Tension provided by the posts of the CFI device was sufficient to induce myofiber alignment, and fibronectin supplementation exhibited a higher degree of myofiber alignment than all other constructs. The device allowed for continuous, non-invasive measurement of tissue compaction force, allowing us to study the relationship between tissue compaction force production and the addition of ECM proteins without disrupting tissue development or sacrificing samples for histology. This allowed us to not only measure tissue compaction force but to also observe how these forces changed as the tissue developed throughout culture. Overall, these results suggest a potential relationship between ECM protein composition, tension, and tissue compaction that may be utilized to produce denser, bundled skeletal muscle tissues capable of treating VML injuries. A limitation of this study is that we focused on bulk construct properties (i.e., passive compaction and structural alignment) and did not directly assess sarcomere organization, adult myosin isoform expression, or active contractile force generation. Future studies will incorporate functional stimulation assays and mechanical characterization of bulk hydrogel stiffness to further delineate biochemical versus mechanical contributions to tissue maturation. While the concentrations identified in this study are specific to the C2C12 model system, the observation of a clear and reproducible relationship between ECM protein composition and whole-tissue compaction suggests that this framework is adaptable to more clinically relevant cell sources. As such, the specific concentrations of fibronectin and laminin identified here may require further optimization prior to application in regenerative contexts. Nevertheless, the replacement of Matrigel with individually characterized ECM components such as fibronectin represents a meaningful step toward reduced reliance on undefined, batch-variable supplements in the fabrication of engineered muscle constructs for eventual clinical translation. Future studies should evaluate the performance of these constructs using primary human cell systems and assess their capacity to generate active contractile force following functional stimulation, which would provide a more direct measure of their potential utility in the treatment of VML injuries.

## Figures and Tables

**Table 1:** Determination of inter-device post modulus and variability. Values are reported as mean ± standard deviation. L: post length; d: post diameter (mid-shaft, measured); Area: cross-sectional area (πd^2^/4); Δθ: angular deflection under load; δ: tip deflection (L·sinΔθ); E: Young’s modulus determined by the Euler–Bernoulli cantilever beam equation.

Device	L (cm)	d (mm)	Area (mm^2^)	Δθ (°)	δ (mm)	E (kPa)	E (MPa)
**Batch 1 (n = 4)**							
Post 1	0.996	0.95	0.709	17.77	3.040	1196.2	1.196
Post 2	0.998	0.92	0.665	29.23	4.873	853.5	0.854
Post 3	1.031	0.94	0.694	18.68	3.303	1273.9	1.274
Post 4	0.959	0.93	0.679	21.07	3.448	1025.0	1.025
**Mean ± SD**	**0.996 ± 0.029**	**0.94 ± 0.01**	**0.687 ± 0.019**	**21.69 ± 5.22**	**3.666 ± 0.822**	**1087.2 ± 187.3**	**1.087 ± 0.187**
**Batch 2 (n = 4)**							
Post 1	1.033	0.94	0.694	22.99	4.035	1048.8	1.049
Post 2	0.981	0.93	0.679	23.18	3.862	979.6	0.980
Post 3	1.010	0.92	0.665	29.10	4.912	877.7	0.878
Post 4	1.003	0.93	0.679	25.35	4.294	941.7	0.942
**Mean ± SD**	**1.007 ± 0.021**	**0.93 ± 0.01**	**0.679 ± 0.012**	**25.16 ± 2.84**	**4.276 ± 0.460**	**962.0 ± 71.6**	**0.962 ± 0.072**
**Overall mean ± SD (N = 8)**						**1024.6 ± 147.3**	**1.025 ± 0.147**

## Data Availability

The original contributions presented in this study are included in the article. Further inquiries can be directed to the corresponding author.

## Abbreviations

The following abbreviations are used in this manuscript:

ECM	Extracellular matrix
VML	Volumetric muscle loss
CFI	Contractile force indicator
PDMS	Polydimethylsiloxane
GM	Growth media
BSA	Bovine serum albumin
DM	Differentiation media
FBS	Fetal bovine serum
PBS	Phosphate-buffered saline
PFA	Paraformaldehyde
OCT	Optimal cutting temperature compound
